# The effect of omega-3 unsaturated fatty acids on non-alcoholic fatty liver disease: A systematic review and meta-analysis of RCTs

**DOI:** 10.12669/pjms.334.12315

**Published:** 2017

**Authors:** Le Yu, Man Yuan, Linchun Wang

**Affiliations:** 1 Dr. Le Yu, Center of Infectious Diseases, West China Hospital, Sichuan University, No. 37 Guoxue Alley, Wuhou District, Chengdu, 610041, Sichuan, China. Division of Infectious Diseases, State Key Laboratory of Biotherapy and Cancer Center, West China Hospital, Sichuan University and Collaborative Innovation Center for Biotherapy, Chengdu, Sichuan, China; 2 Dr. Man Yuan, Center of Infectious Diseases, West China Hospital, Sichuan University, No. 37 Guoxue Alley, Wuhou District, Chengdu, 610041, Sichuan, China. Division of Infectious Diseases, State Key Laboratory of Biotherapy and Cancer Center, West China Hospital, Sichuan University and Collaborative Innovation Center for Biotherapy, Chengdu, Sichuan, China; 3 Linchun Wang, Center of Infectious Diseases, West China Hospital, Sichuan University, No. 37 Guoxue Alley, Wuhou District, Chengdu, 610041, Sichuan, China. Division of Infectious Diseases, State Key Laboratory of Biotherapy and Cancer Center, West China Hospital, Sichuan University and Collaborative Innovation Center for Biotherapy, Chengdu, Sichuan, China

**Keywords:** Meta-analysis, Non-alcoholic fatty liver disease, Omega-3 unsaturated fatty acids

## Abstract

**Objective:**

During the treatment of diseases such as angiocardiopathy, blood lipid abnormalities and metabolic syndrome, omega-3 unsaturated fatty acids (PUFA) can reduce plasma lipids and improve cardiovascular status, thus ameliorating disease severity. We aimed to explore the effects of PUFA supplementation in patients with non-alcoholic fatty liver disease (NAFLD).

**Methods:**

A systematic literature search was performed during March 2016 for randomized controlled trials using PUFA or fish oil supplementation in patients with NAFLD or non-alcoholic steatohepatitis (NASH). All Randomized controlled trials were retrieved from MEDLINE and EMBASE database up to date (March 2016). A meta-analysis of key outcomes (serum level of liver enzymes and lipids) were identified in these studies. The mean difference (MD) and the corresponding 95% confidence intervals (CIs) were used as measures of effect size.

**Results:**

Thirteen studies were included, consisting of 266 patients in the PUFA group and 402 cases in the control group. Serum level of alanine aminotransferase (ALT) was lower in the PUFA group than that in in the controls [MD=−9.18, 95% CI (−12.41, −5.96), *P* <0.00001]. However, PUFA treatment did not affect aspartate aminotransferase (AST) [MD=−5.07, 95% CI (−12.65, 2.51), P= 0.19], gamma-glutamyl transferase (GGT) [MD=−1.91, 95% CI (−4.15, 0.33), *P* <0.009].

**Conclusions:**

PUFA supplementation may affects serum level of ALT and improve liver function in patients with NAFLD.

## INTRODUCTION

Non-alcoholic fatty liver disease (NAFLD) is a clinicopathological syndrome characterized by excess deposition of fat in the hepatocytes that is associated with risk factors other than excess alcohol intake. It refers to a spectrum of pathological changes in the liver, including non-alcoholic steatohepatitis (NASH). If left untreated, liver cirrhosis or liver cancer can subsequently develop.^1^,^2^ The current global morbidity of NAFLD has been estimated to be 20%–33%, reflecting its status as one of the most common liver diseases in Western countries. Although NAFLD morbidity is lower in China, it is approximately 15% and rising annually in well-off regions of the country.^3^-^6^ Although the pathogenesis of NAFLD has not been fully characterized, its occurrence and severity are closely linked with high blood pressure, insulin resistance, obesity and dyslipidemia.^7^,^8^

Although there are no effective specific treatments available for NAFLD, exercise and dietary therapy play important roles in its treatment.^9^,^10^ Previous studies have shown that exercise can improve plasma lipid profile and liver function, thus delaying the progress of disease.^11^ In addition, dietary therapy based on vitamin E supplementation has been proposed for the treatment of liver cirrhosis.

Recent research has suggested that ω-3 unsaturated fatty acid (PUFA) administration improves plasma lipid profile and may be useful in the treatment of NAFLD.^12^-^14^ A previous meta-analysis of PUFA data derived from clinical trials confirmed an improvement in patients with NAFLD.^15^ However, this analysis included clinical trials other than purely randomized controlled trials (RCTs). In the present study, we have collected and systematically reviewed the published results of RCTs, and we further discuss the utility of PUFA as a treatment for NAFLD patients and aim to integrate the existing data, providing a suggestion for the possible clinical practice of PUFA.

## METHODS

### Literature Search Strategy

A systematic literature search was undertaken during March 2016 for publications describing clinical studies using PUFA or fish oil supplementation for the treatment of patients with NAFLD or NASH. PubMed was used to search the MEDLINE database and Ovid and Clinical Key were used to search the EMBASE database. The search terms used were: ‘fish oil’, ‘EPA’, ‘eicosapentaenoic acid’, ‘eicosapentaenoic acid’, ‘DHA’, ‘docosahexaenoic acid’, ‘docosahexaenoic acid’, ‘omega-3’, ‘PUFA’, ‘n-3’, AND ‘NAFLD’, ‘hepatic steatosis’, ‘NASH’. We also manually searched for additional relevant studies given in the reference lists of review articles identified through these databases. Each retrieved abstract was independently reviewed by two authors and a decision on its inclusion or exclusion was made. For each study included, an additional researcher obtained the full text article for further review. Disagreements were resolved by discussion among the three authors.

### Study Selection Criteria

We included publications of RCTs using DHA, EPA, fish oil, or other PUFA therapy in male or female patients with advanced NAFLD or NASH. Studies were included if they contained patients diagnosed with NAFLD/NASH alone or if the patients had been diagnosed with other diseases in addition to NAFLD/NASH. Studies of both children and adults were included. Studies were excluded if they included patients with alcoholic liver disease, viral hepatitis, haemochromatosis, Wilson’s disease, autoimmune hepatitis, liver disease caused by drugs or toxins, or other concurrent disease that might have influenced our analysis. We also excluded studies if important information was not provided or if the data had been reported more than once in the literature.

### Data Extraction

According to the original literatures, we used serum alanine aminotransferase (ALT), glutamic-oxaloacetic transaminase (AST), gamma-glutamyl transferase (γ-GGT), triglycerides, fasting glucose, high density lipoprotein (HDL) and low density lipoprotein (LDL) as the outcome measures in the analysis. Basic information regarding the patients, interventions used, adverse events recognized, cohort standardization and timing were also extracted. Data were collated in a predefined format to ensure comparable classification of each variable. In addition, all data were validated by comparing the outcome in the original publications, and discrepancies were resolved by discussion and by contacting the authors if necessary.

### Quality assessment

Evaluation of the methodological quality of each clinical trial was undertaken independently by two authors, and differences in the opinion were resolved by discussion, with a third author if necessary. Quality was evaluated using the Jadad quality score, which is a five-point scale. Studies awarded one or two points were categorized as low quality research, while studies awarded 3 to 5 points were categorized as the high quality research. Under the following circumstances, the study was awarded zero points: semi random design (alternating distribution of cases, such as according to the order of admission or date of birth), lack of double- blind method or no mention of the use of double-blind test, or no mention of withdrawal or loss to follow-up of study patients.

### Statistical analysis

All variables in the included studies were continuous; the pooled estimate of the mean difference (MD) and the 95% confidence interval (CI) were used to analyze the effect size of the studies. Statistical heterogeneity among individuals was evaluated using Cochran’s χ^2^ test and the I^2^ statistic. A fixed effects model was used to pool the results where statistical homogeneity (P >0.1, <50% I^2^) was observed and a random effects model was used if statistical heterogeneity (P <0.1, I^2^ >50%) was present. When heterogeneity was found in the included studies, efforts were made to identify the source, and sensitivity analysis was carried out if the heterogeneity was derived from low quality studies or contrasting evaluation methods. Statistical analysis was conducted using Review Manager 5.3 (The Nordic Cochrane Centre and the Cochrane Collaboration, Copenhagen, Denmark, 2014).

## RESULTS

**Fig. 1:**
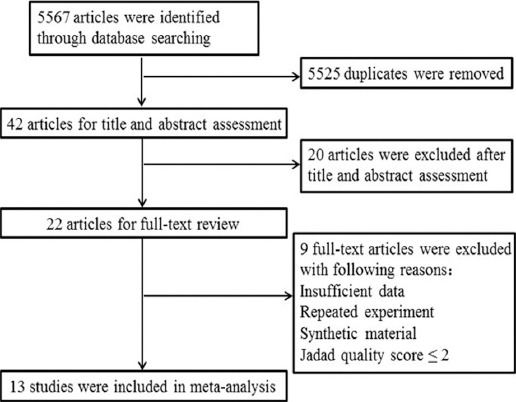
Flow-chart identifying eligible studies.

Total 5567 articles were retrieved using the search terms. Examination of the title and abstract permitted exclusion of non-clinical laboratory studies, animal experiments, and studies published in more than one place and those that obviously did not meet the inclusion criteria, leaving 22 papers left. After reading the full text, nine studies were identified that did not meet the inclusion criteria, leaving 13 studies, of which 12 were written in English and one in Chinese^16^-^28^ (Fig. 1). These studies reported data for a total of 843 patients, which were used in our analysis. There were contained 266 patients (301 males and 175 females) in the PUFA group and 402 patients (245 males and 157 females) in the control group. All studies were assigned a Jadad score of ≥3, while five studies received a score of <4. All of the seven included studies were stated to be randomized (Table-I, Table-II).

**Table-I:** Summary of included studies in meta-analysis.

*Authors*	*Year*	*Location*	*Intervention*		*Sample (size/female)*	*Duration of intervention*

			*Intervention group*	*Control group*		
Spadaro et al.	2007	Italy	PUFAs	Placebo	36/17	6 months
Zhu et al.	2008	China	PUFA	Placebo	134/37	6 months
SOFI et al.	2010	Italy	EPA DHA	Olive oil	11/2	12 months
Argo et al.	2014	America	PUFA	Soybean oil	34/21	12 months
Boyraz et al.	2015	Turkey	PUFA	Placebo	108/53	12 months
Li et al.	2015	China	EPA DHA	Normal saline	78/8	6 months
Dasarathy et al.	2015	America	EPA DHA	Corn oil	37/29	12 months
McCormick et al.	2015	UK	EPA DHA	Olive oil	103/43	18 months
Chen et al.	2008	China	EPA DHA	Normal saline	46/16	6 months
V. Nobili et al.	2012	Italy	DHA	Placebo	60/35	24 months
Qin et al.	2015	China	fish oil	Corn oil	70/19	3 months
Janczyk et al.	2015	UK	PUFA	Omega-6 sunflower oil	76/11	6 months
Nogueira	2015	Brazil	ALA+EPA+DHA	Mineral oil	50/41	6 months

**Table-II:** Characteristics of included patients in meta-analysis.

*Authors*	*Sample(size/female)*		*Age*		*BMI*	

	*W-3*	*Control*	*W-3*	*Control*	*W-3*	*Control*
Spadaro et al.	18/7	18/10	50.16±12.9	51.3±9.8	30.1±4.7	31.0±3.4
Zhu et al.	66/19	68/18	45.00± 10.91	44.03± 11.30	26.37±3.12	25.96 ± 2.70
SOFI et al.	6/2	5/0	55 (30–41)	54 (42–70)	29.3±4.1	29.3±3.9
Argo et al.	17/10	17/11	46.4±12.1	47.2±12	33.3±8.0	31.6±6.7
Boyraz et al.	56/29	52/24	13.8±3.8	13.3±3.5	29.7±4.8	27.2±3.3
Li et al.	39/3	39/5	52.6±6.6	50.4±7.2	28.0±1.4	27.2±1.3
Dasarathy et al.	18/12	19/17	51.5±6.9	49.8±12.1	34.8±4.6	35.7±7.0
McCormick et al.	51/26	52/17	48.6±11.1	54.0±9.6	34.3±5.8	32.0±4.3
Chen et al.	30/6	16/10	46.5	45		
V. Nobili et al.	40/23	20/12	11(8-14)	13(9-17)	26.6 (21.7-31.5)	26.1(21-31.2)
Qin et al.	36/10	34/9	46.0±10.68	44.3±10.90	26.4±3.9	26.0±2.8
Janczyk et al.	37/5	39/6	13.2 (11.2-15.9)	12.8 (10.9-14.9)	28.6 (26.3-32.1)	28.86 (25.6-32.0)
Nogueira et al.	27/23	23/18	52.5 ± 7.2	53.9 ± 6.8	31.1 ± 4.6	30.3 ± 4.4

BMI: body mass index, Data are expressed as the mean ± standard deviation, or median and interquartile range (IQR).

### Effect of PAFU on the AST change after treatment

**Fig. 2:**
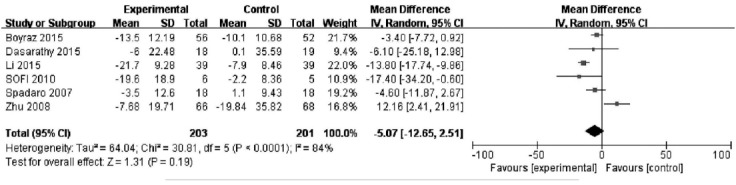
Effect of PAFU on the AST change after treatment.

Ten studies reported measurements of AST in PUFA and control groups; however, four of these studies did not provide a standard deviation, therefore six studies (n=404) were used in the analysis. The assessment of heterogeneity produced *P* <0.0001 in Cochran’s Q test and I^2^= -84%, which indicated that there was no significant variability between these studies. And therefore a random effects model was used. There was no significantly difference in serum level of AST between the groups [MD=−5.07, 95% CI (−12.65, 2.51), *P* =0.19]. (Fig. 2)

### Effect of PAFU on the ALT change after treatment

**Fig. 3:**
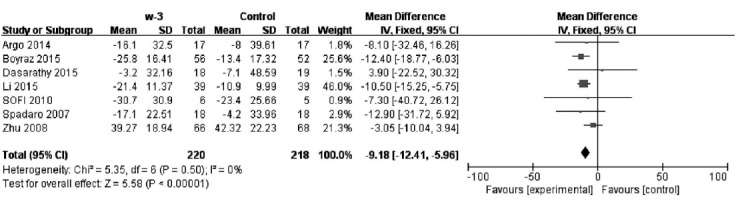
Effect of PAFU on the ALT change after treatment.

Nine studies reported measurements of ALT in PUFA and control groups; however, two of these studies did not provide a standard deviation, therefore seven studies (n=438) were used in the analysis. There was no significant heterogeneity (*P*= 0.50; I2= 0%) and the fixed effects model was therefore used. There was a significant difference in ALT between the groups [MD=−9.18, 95% CI (−12.41, −5.96), P<0.00001]. These data are consistent with PUFA treatment leading to a decrease in ALT in NAFLD patients. (Fig. 3)

### Effect of PAFU on the GGT change after treatment

**Fig. 4:**
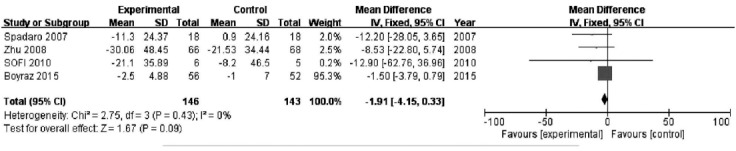
Effect of PAFU on the GGT change after treatment.

Seven studies reported measurements of γ-GGT in PUFA and control groups; however, three did not provide a standard deviation, so four studies (n=289) were used in the analysis. There was no significant heterogeneity (*P*= 0.43; I^2^ = 0%) and therefore a fixed effects model was used. γ-GGT was not significantly different between the two groups [MD=−1.91, 95% CI (−4.15, 0.33), P=0.009]. (Fig. 4)

### Effect of PAFU on the other changes after treatment

Original researches included also mentioned some other parameters’s change in different groups, including Triglycerides, Fasting glucose, HDL and LDL, and six(n=401), five (n=268), seven(n=507) and six researches (n=468) were taken into analysis these parameters, respectively. Our results showed that Triglycerides [MD=-29.07, 95%CI(-48.22,-9.91), P<0.003], HDL [MD=4.81, 95%CI(1.59, 8.03), P=0.03], LDL [MD=-9.18, 95%CI(-14.89, -3.47), P<0.002] in the two groups were statistically significant, however, the Fasting glucose of patients were not significantly different in the two groups [MD=-0.09, 95%CI(-2.54, 2.72), P<0.95]. The treatment of fatty acid decreased Triglycerides and LDL and increased the level of HDL in NAFLD patients at the same time (Table-III).

**Table-III:** Triglycerides, Fasting glucose, HDL and LDL changes in different groups.

*Index*	*Sample*		*Heterogeneity*		*Mean difference*		

	*W-3*	*Control*	*P*	*I^2^*	*MD*	*95%CI*	*P*
Triglycerides	202	199	0.04	57%	-29.07	-48.22,-9.91	0.003
Fasting glucose	136	132	0.42	0%	-0.09	-2.54, 2.72	0.95
HDL	254	253	0.009	65%	4.81	1.59, 8.03	0.03
LDL	235	233	0.43	0%	-9.18	-14.89, -3.47	0.002

## DISCUSSION

PUFA, a group of unsaturated aliphatic fatty acids, includes α-linolenic acid, eicosapentaenoic acid (EPA) and docosahexaenoic acid (DHA). Previous research has suggested that PUFA may influence lipid metabolism by regulating hepatic gene transcription. Specifically, PUFA affect transcription of the peroxisome proliferator-activated receptor, sterol regulatory element binding protein-1 and the carbohydrate regulatory element gene. Because these transcription factors are key determinants of lipid and carbohydrate metabolism, the synthesis, metabolism and elimination of hepatic lipids, resulting in altered hepatic lipid storage levels.^29^ In addition, PUFA can also reduce the activity of enzymes, increase lipoprotein breakdown and reduce plasma lipid content, resulting in the reduced uptake of lipid by hepatocytes. Furthermore, animal experiments have shown that PUFA can protect the liver from fatty degeneration.

In recent years, many studies have been undertaken to establish whether PUFA supplementation might be beneficial for human disease. PUFA can reduce plasma lipid content and improve cardiovascular status, providing potential effective therapeutic agents for use in patients with angiocardiopathy, blood lipid abnormalities and metabolic syndrome.^13^,^30^

Owing to the lipid-lowering effects of PUFA in the treatment of the above diseases, a beneficial effect has been proposed for patients with NAFLD. RCTs have shown that PUFA supplementation might reduce serum level of ALT, AST and lipid content in NAFLD patients. However, the number of patients involved in these trials was relatively small and further induction analysis was not undertaken. Our meta-analysis included only RCTs trails and confirmed that PUFA had an impact on NAFLD patients, indicated by changes of clinical parameters.

The results of our meta-analysis are not identical to those of the meta-analysis of Parker et al^15^, which showed a significant benefit of PUFA therapy on AST and a tendency toward a benefit on ALT. However, in contrast to our meta-analysis, previous analysis included studies other than RCTs, making the interpretation of the findings less reliable, and it also pointed out that AST and ALT changes were not significant after examination of only RCT data. As mentioned by Parker, the high intra-individual variability in liver function tests may influence the result, and randomized controlled trials with large sample were required for better understanding of this therapy. Our study only systematically reviewed the published results of RCTs, and more researches updated in recent years had taken consideration. More high quality data showed the ability of PUFA on the effect of ALT. However, there is still a lack of strong evidence for a PUFA-related improvement in AST and γ-GGT in NAFLD patients.

There were a number of deficiencies identified in the publications, which were used for our meta-analysis that hamper its interpretation. Some instances showed that, there were no reports on serious adverse events associated with the administration of PUFA. In addition, varying quantities and types of PUFA were used in the RCTs examined, so that no effect of dose on study outcome could be analysed. The effects of PUFA dose on study outcome were examined in only two studies. In a study from China, 46 adult patients were randomly allocated to a high dose group (1250mg/d), a low dose group (1000mg/d) and a placebo group and treated for 24 weeks.^24^ The improvement in the ultrasound score of liver was in a PUFA dose-dependent manner and there were also lower serum triglycerides in the higher dose group, suggesting a higher efficacy of the 1250mg/d regimen for the treatment of NAFLD. Sixty children were included in the study by Nobili et al.^31^; who were randomly allocated to control, high dose (500mg/d DHA) and low dose (250 mg/d DHA) groups, and treated for 24 months. In this study, however, although DHA was effective in treating the NAFLD patients, there was no apparent dose-dependency on the effect.

There was a range of criterias applied to the analysis of imaging results in the various studies and consequently our meta-analysis did not incorporate imaging data. There were some inconsistencies between studies regarding the effect of PUFA on radiological changes in patients PUFA could significantly improve the B-ultrasound score in NAFLD patients,^24^ another study did not show significant improvement in liver histopathology after PUFA treatment.^23^ Such variation may be the result of insufficient study duration; it is likely that recognition of radiological improvement in patients requires a longer study period. Thus, further longer-term studies of larger numbers of subjects may be helpful to accurately determine the effects of PUFA on the progression of radiological changes of the patients.

Our study, also showed that the patients’ HDL and LDL were significantly changed after more than one year PUFA treatment, and we also find that the effect on HDL was not statistically significant after six months PUFA treatment. These data demonstrated that a therapeutic effect of PUFA may require long-term administration. It is therefore important to note that among the included studies, many were of less than one year’s duration. Further analysis of the effect of treatment duration and the side effects will be undertaken as a follow-up.

In our meta-analysis we have considered the effect of PUFA as a group and not those of individual species, such as AHA and DHA. Among the incorporated studies, combined treatments with EPA and AHA were widely used and most of the other studies also used a mixture of PUFA (see Table-II). Thus, further studies are necessary to identify the effects of individual PUFA.

In conclusion, the present meta-analysis suggests that PUFA may affect serum level of ALT and thereby improve liver function in patients with NAFLD. Further large scale RCTs, incorporating biochemical and histological outcome measures, are needed to explore their therapeutic utility.

### Authors Contribution

**LY** conceived, designed and did data collection, statistical analysis & manuscript writing.

**MY** did editing of manuscript.

**LW** did review and final approval of manuscript.
